# The Effectiveness of Bacteriophages against Methicillin-Resistant *Staphylococcus aureus* ST398 Nasal Colonization in Pigs

**DOI:** 10.1371/journal.pone.0160242

**Published:** 2016-08-03

**Authors:** Koen M Verstappen, Pawel Tulinski, Birgitta Duim, Ad C Fluit, Jennifer Carney, Arie van Nes, Jaap A Wagenaar

**Affiliations:** 1 Department of Infectious Diseases and Immunology, Faculty of Veterinary Medicine, Utrecht University, Utrecht, the Netherlands; 2 Department of Medical Microbiology, University Medical Centre Utrecht, Utrecht, the Netherlands; 3 Novolytics Ltd., Warrington, United Kingdom; 4 Department of Farm Animal Health, Faculty of Veterinary Medicine, Utrecht University, Utrecht, the Netherlands; 5 Central Veterinary Institute of Wageningen UR, Lelystad, the Netherlands; University of Minnesota, UNITED STATES

## Abstract

Methicillin-resistant *Staphylococcus aureus* (MRSA) is an important colonizer in animals and an opportunistic pathogen in humans. In humans, MRSA can cause infections that might be difficult to treat because of antimicrobial resistance. The use of bacteriophages has been suggested as a potential approach for the control of MRSA colonization to minimize the—often occupational—exposure of humans. The aim of this study was to assess the efficacy of bacteriophage treatment on porcine nasal colonization with MRSA *in vitro*, *in vivo*, and *ex vivo*. The effectiveness of a bacteriophage combination of phage K*710 and P68 was assessed *in vitro* by incubating them with MRSA V0608892/1 (ST398) measuring the OD_600_ hourly. To study the *in vivo* effect, bacteriophages were administered in a gel developed for human application, which contain 10^9^ plaque-forming units (pfu)/mL (K and P68 in a 19.25:1 ratio) for 5 days to piglets (N = 8) that were experimentally colonized with the MRSA strain. Eight piglets experimentally colonized were used as a negative control. The MRSA strain was also used to colonize porcine nasal mucosa explants and bacteriophages were applied to assess the *ex vivo* efficacy of treatment. Bacteriophages were effective *in vitro*. *In vivo*, sixteen piglets were colonized with MRSA but the number of CFU recovered after the application of the bacteriophages in 8 piglets was not reduced compared to the control animals (approx. 10^5^ CFU/swab). In the *ex vivo* model, 10^8^ CFU were used to establish colonization with MRSA; a reduction of colonization was not observed after application of bacteriophages. However, application of mupirocin both *in vivo* and *ex vivo* resulted in a near eradication of MRSA. In conclusion: i) The MRSA strain was killed in the presence of the bacteriophages phage K*710 and P68 *in vitro*. ii) Bacteriophages did not reduce porcine nasal colonization *in vivo* or *ex vivo*. Physiological *in vivo* and *ex vivo* conditions may explain these observations. Efficacy in the *ex vivo* model matched that of the *in vivo* system.

## Introduction

Worldwide, methicillin-resistant *Staphylococcus aureus* (MRSA) is an important colonizer in animals and an opportunistic pathogen in humans. During the last decade Livestock-Associated (LA-) MRSA of sequence type (ST) 398 has emerged in Europe and North America [1–4]. The transmission of MRSA ST398 from livestock to humans has been reported in many countries [5,6] and contact with livestock is recognized as a risk factor for the presence in humans [7,8]. Although this type of MRSA is believed to have adapted to livestock, ST398 is able to colonize and cause infections in humans [9,10]. Reduction of prevalence of colonized livestock or reduction of shedding by positive animals will reduce exposure, and thus the presence of LA-MRSA in humans. Other options than antimicrobial chemotherapeutic agents should be explored for efficacy to reduce LA-MRSA in animals, because antimicrobial use in animals for the purpose of decolonization is a highly unwanted situation.

Bacteriophage therapy offers a possible alternative to classic antibiotic (antimicrobial chemotherapeutic) treatment to reduce bacterial colonization [11]. Bacteriophages are able to infect bacteria and enter either a lysogenic or a lytic cycle, with infection by constitutively lytic bacteriophages generally resulting in rapid cell death. Also, if the phage-to-cell ratio is high enough, lysis from without may cause the cell to burst before infection is initiated [12]. A main advantage of bacteriophages is their specificity. Whereas classic therapeutic treatment with chemical antimicrobials affects many different organisms in the body (e.g. the gut microbiota), causing a change in the microbial composition and inducing antimicrobial resistance in a spectrum of bacterial species, bacteriophages are able to specifically target the organism or even only the strain that is causing the infection. The *in vitro* lytic effect of bacteriophages can be easily tested. However, the use of bacteriophages for therapy (e.g. to treat infections or reduce colonization) poses additional challenges like the accessibility of bacteria and *in vivo* inactivation of bacteriophages.

The aim of this study was to assess the effectiveness of bacteriophage treatment on porcine nasal colonization with MRSA using *in vitro*, *in vivo* and *ex vivo* models.

## Materials and Methods

### Bacteria and bacteriophages

MRSA strain V0608892/1 (ST398, *spa* type t011, SCC*mec* type V) was used for these experiments. The strain was isolated from a healthy pig upon diagnostic screening.

A bacteriophage solution in a proprietary gel formulation developed for human application by Novolytics Ltd. (United Kingdom), containing bacteriophages P68 (a podovirus) [13] and phage K*710 (a myovirus) [14] in a 1:19.25 ratio with a final concentration of 10^9^ plaque-forming units (pfu)/mL was used. The phages were also tested in a liquid solution but were less effective than in gel (data not shown). Both phages were propagated on MRSA strain SAI653 and are effective against several MRSA isolates of ST398 from pigs in the Netherlands and Denmark (data not shown). Both phages were propagated on MRSA strain SAI653 and are effective against a broad range of isolates including several MRSA isolates of ST398 from pigs in the Netherlands and Denmark (S1 Table). The gel formulation without bacteriophages was used as a placebo control.

### *In vitro*: growth curves

To evaluate the *in vitro* effectiveness of the bacteriophages the MRSA strain was grown overnight in BHI (Oxoid, the Netherlands) at 37°C. A 1:50 dilution was prepared in fresh BHI and cells were grown for approx. 3 h to mid-exponential phase at 37°C with shaking at 200 rpm. The cell suspension was diluted in BHI to a concentration of approximately 10^7^ CFU/mL based on optical density at 600 nm (OD_600_). A volume of 380 μL was transferred to an optical multi-well plate in duplicate and 20 μL bacteriophage-containing gel (multiplicity of infection, MOI 5.3) or 20 μL placebo was added. The plate was incubated in a BioScanner C (Labsystem France SA, France) at 37°C for 20 h; OD_600_ was measured every hour and the experiment was performed three times.

### *In vivo*: piglets colonization

Sixteen crossbred, caesarean derived colostrum deprived (CD/CD) crossbred piglets (obtained from two different sows) were equally divided over 4 isolators (A-D). Animals were obtained, housed and fed [15] and colonization was established [16] as previously described. In brief, colonization with MRSA was established as follows: at the age of 5 days the animals were screened for the absence of MRSA and at the age of 6 days the animals received 500 μL intranasal inoculation of 10^9^ CFU/animal with strain V0608892/1. The strain was grown to log-phase in BHI broth, washed with PBS and adjusted based on cell density. Numbers of MRSA were monitored by nasal sampling at days 7, 8, 9, 11 and 12; samples were quantitatively analysed for the presence of MRSA on Brilliance MRSA 2 Agar (Oxoid, the Netherlands) as described before [16]. Numbers of MRSA are reported as CFU/swab.

Five hundred μL of bacteriophage-containing gel was administered in each nostril to the animals in isolators A and C at the age of 12 days using a syringe. The animals in isolators B and D received a placebo (identical gel without the bacteriophages). These treatments were performed daily for 5 days. Nasal samples were obtained before the administration of bacteriophage or placebo gel on the respective day to monitor colonization. Monitoring continued for one week after the last administration. All samples were processed within 2 h after collection and MRSA was enumerated by quantitative plating as described above.

At day 19 nasal samples of bacteriophage-administered piglets were also analysed for the presence of bacteriophages. After bacterial enumeration samples were passed through a 0.45 μm filter (Pall, the Netherlands). This filtrate of the swab suspension was plated onto Tryptone Soy agar (TSA, Oxoid, the Netherlands) with 1.5% agar, containing strain SAI653. Plates were incubated overnight and assessed for the presence of plaques. If plaques were observed bacteriophages were regrown and, after filtration, stored in 50% glycerol at -80°C. To test if the bacteriophages were still effective against the bacteria colonizing the piglets, the original gel suspension, 10 μL filtrate of the swab suspension (day 19), as well as this suspension after storage at -80°C were spotted onto TSA with the bacterial isolate from the respective pig at 16 days.

In order to show that a reduction of colonization could be achieved the animals in isolators A and B were intranasally treated with a mupirocin ointment (Bactroban^®^ 2%) at the age of 22 days for the duration of 5 days, 2 daily doses of 50 mg; groups C and D remained untreated (no placebo was administered). Nasal swabs were obtained at days 27, 28, 29, and 30 for enumeration of MRSA to study the effect of mupirocin treatment.

### *Ex vivo*: nasal mucosa colonization

To evaluate the effectiveness of the bacteriophage-containing gel on mucosal explants, porcine tissue was obtained and the colonization assay was performed as described previously with some modifications [17]. In brief, after nasal mucosa membrane isolation the stripped mucosa tissue was divided into explant pieces of 0.5 cm^2^ using 8 mm biopsy punches (AcuDerm Inc, USA) and incubated at an air-liquid interface. The explants were colonized with a bacterial inoculum, approximately 2×10^8^ CFU in 1 mL Dulbecco’s PBS with magnesium and calcium (DPBS; Gibco, the Netherlands), for 2 h to allow the bacterial adhesion to the tissue. Next, explants were washed three times with 1 mL DPBS. The time-point for this post-adhering-step is defined as t = 0. The inoculated explants were incubated for 1 h [17]. Then, bacteria were isolated from the explants by scraping the epithelium surface using cell scrapers (Falcon, Becton Dickinson, the Netherlands), and cells were suspended in 1 mL DPBS with 0.1% Triton X-100 (t = 1; measurement of initial attachment of bacteria). Bacterial suspensions were plated on Columbia agar with sheep blood (Oxoid, the Netherlands) in serial dilutions (1:10) in DPBS. The plates were incubated overnight and bacteria were enumerated. All incubation steps of the explants were performed at 37°C and 5% CO_2_ atmosphere, incubation of bacteria was performed at 37°C under aerobic conditions.

To evaluate the effectiveness of the bacteriophages, 50 μL bacteriophage-containing gel (MOI 10) or placebo gel was applied after 1 h of colonization (t = 1). Additionally, 50 μL mupirocin (Bactroban^®^ 2%) solution (1:1 in DPBS) was applied to another set of explants. To control for the growth of *S*. *aureus* on the explants the last set of explants was not treated. MRSA was enumerated 4 h (t = 4) and 24 h (t = 24) post-adhering as described above. The experiment was performed 3 times independently.

## Statistics

All numbers of CFUs were log-transformed. Mean concentrations and standard deviations were calculated from replicates (*in vitro* and *ex vivo* experiments). In the *in vivo* experiment each isolator (containing 4 piglets) was considered to be one experimental unit and thus the mean concentrations and standard deviations were calculated per isolator.

The concentrations of MRSA on nasal mucosa explants were compared using an analysis of variance (ANOVA) to evaluate the effectiveness of the bacteriophage-containing gel and mupirocin application. The concentrations of MRSA in nasal samples were compared between isolators by ANOVA to see the effect of the bacteriophage treatment (days 13–22) and the mupirocin ointment (days 27–30).

## Ethics

These experiments were approved by the Animal Ethical Committee of Utrecht University and were filed under entries 2011.II.11.180 and 2012.II.08.127.

## Results

### In vitro

**Fig 1:**
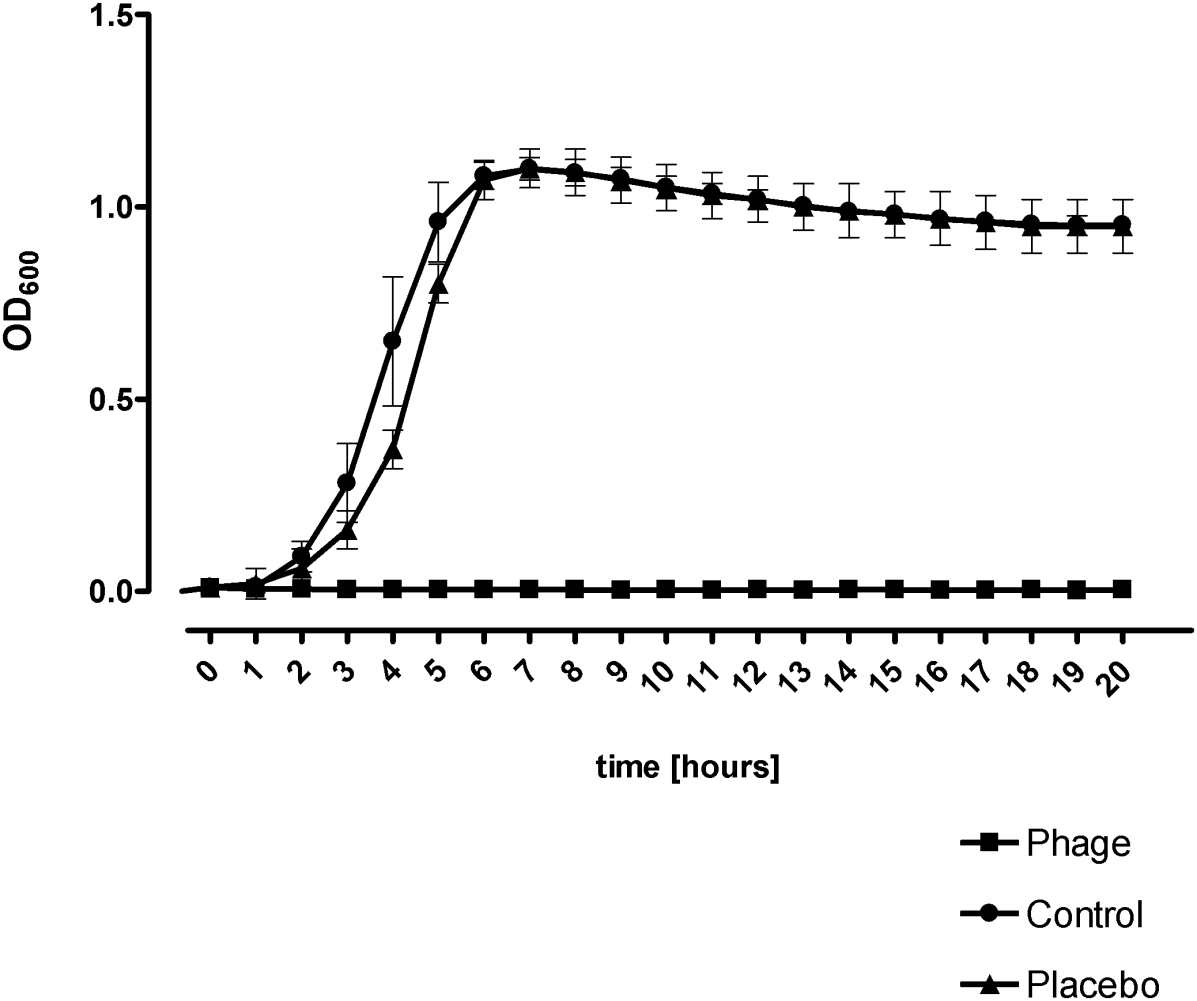
Effect of bacteriophage solution on the growth of MRSA V0608892/1. OD values reflect bacterial concentration in presence of the bacteriophage solution, placebo and no-treated control. The results are presented as the mean OD ± standard deviation of 3 different experiments in duplicate.

An *in vitro* setup was used to evaluate the effectiveness of the bacteriophage-containing gel on MRSA growth in BHI medium (Fig 1). The results showed that the bacteriophage-containing gel prevented bacterial growth, as growth was only observed in the control and placebo samples, where the bacteria reached stationary phase with an OD_600_ of 1.2 within 5 h. Additionally, no significant differences between placebo and control samples were observed (Fig 1).

### In vivo

**Fig 2:**
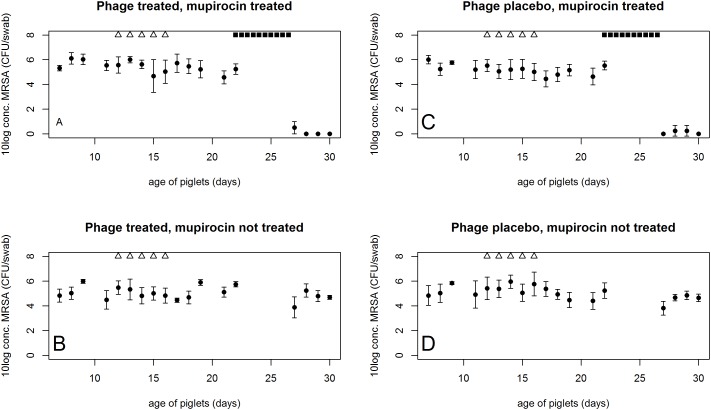
MRSA recovery in the *in vivo* experiment. Culture results for nasal samples in the *in vivo* experiment. Numbers of MRSA are displayed in CFU/swab. The error bars represent standard deviation. Each group consisted of 4 piglets. Group A) piglets that were treated with bacteriophage solution and received mupirocin ointment; B) piglets that received a placebo without bacteriophage and were treated with mupirocin ointment; group C) received a bacteriophage treatment, but no mupirocin and group D) was administered a placebo without bacteriophage and was not treated with mupirocin. △) indicate bacteriophage or placebo treatment; ■) indicate ointment with mupirocin. If a sample was obtained on the same day as treatment took place the sample was taken before treatment was applied.

The bacteriophage-containing gel prevented bacterial growth *in vitro*. Therefore, the bacteriophages were tested in an *in vivo* experiment. The MRSA strain could be retrieved from the pigs at numbers varying between 10^4^ and 10^6^ CFU/swab and numbers of MRSA continued to vary between these values throughout the experiment (average of 5.7×10^5^ CFU/swab), see Fig 2 for observations. During and after the application of bacteriophages the numbers of MRSA continued to oscillate between 10^4^−10^6^ CFU/swab in the bacteriophage-treated and placebo-treated pigs. No statistically significant effect was observed during or after bacteriophage treatment (P>0.05, ANOVA).

Bacteriophages were re-isolated from 5/8 piglets at day 19 (3 days after the last bacteriophage treatment) using SAI653 as a culture strain. However, no plaques were observed when these suspensions were plated onto the re-isolated strains from the same piglet at 16 days. The original bacteriophage solution was still effective against these bacterial re-isolates. After enrichment and storage at -80°C bacteriophages could be regrown on the same re-isolated strains (16 days).

Mupirocin treatment reduced the recovery of the inoculated strain. In most samples the inoculated strain could not be detected (P<0.01, ANOVA). Only in three samples from animals in both isolators it was possible to isolate MRSA after enrichment culture (isolator A, day 27; isolator B, day 28 and day 29).

### Ex vivo

Using porcine nasal mucosa explants it was possible to investigate the activity of the bacteriophage-containing gel in a controlled setting that mimicked the *in vivo* situation.

**Fig 3:**
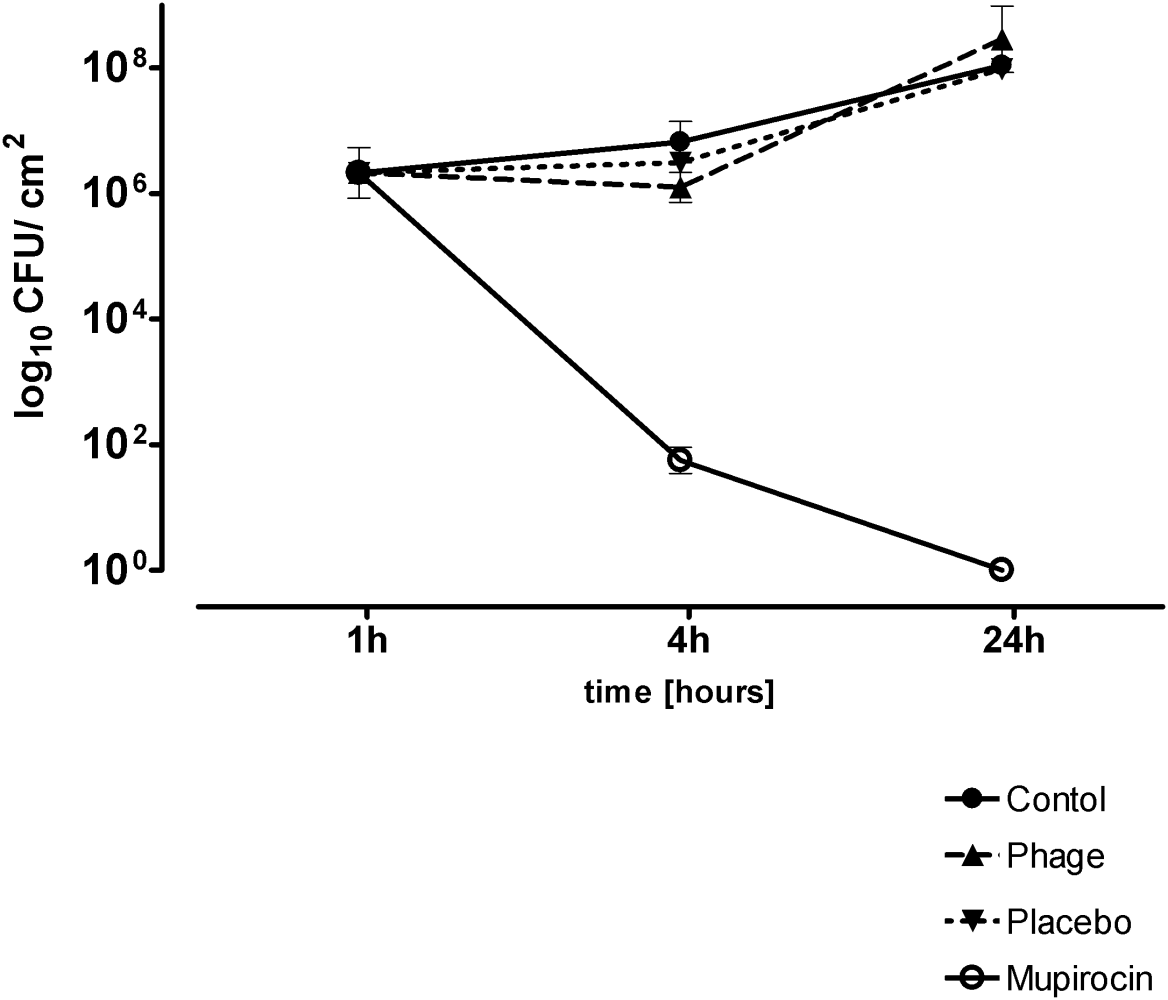
Eradication of MRSA V0608892/1 from the explants after application of bacteriophage solution and mupirocin. Data are presented as the mean CFU ± standard deviation of three experiments; time is hours after adhesion.

Similar to the animal study application of the bacteriophage-containing gel showed no statistically significant differences between control, bacteriophage and placebo treatment (P>0.05, ANOVA). A reduction of colonization of MRSA V0608892/1 from the explants was observed only when mupirocin was applied (P<0.01, ANOVA) (Fig 3).

## Discussion

As human MRSA carriers have an increased risk for MRSA infection, control of MRSA among livestock is important to reduce the exposure and risk for humans [18]. The use of antimicrobial chemotherapy to reach this goal is not desirable because it would select for antimicrobial resistance. An alternative would be the use of bacteriophages. In this study the possibility of bacteriophage treatment to reduce levels of nasal colonization was investigated.

Bacteriophages were able to prevent bacterial growth *in vitro*. However, an effect of the bacteriophages in the *in vivo* and *ex vivo* experiments could not be observed. This indicates that the nasal mucosa explant model can be applied as a preliminary screening for the effectiveness of bacteriophages before *in vivo* experiments are commenced.

It has been reported that an optimal MOI for *in vivo* application of bacteriophages is between 1 and 10 [12]. The MOI in the *ex vivo* experiment was 10, which was in agreement with the suggested MOI. In addition to MOI, an alternative representation of bacteriophage infectivity on a given strain is the efficiency of plating (EOP) as this provides a quantitative assessment [19]. Although the *in vitro* experiments showed promising results, the EOP indicated that one of the two bacteriophages (Phage K*710) had a 10^7^-fold reduced ability to infect strain V0608892/1, when compared to strain SAI653. The ratio of K*710 to P68 in the cocktail was 19.25:1, resulting in an actual effective MOI of 0.52, which was solely caused by phage P68. The bacterial numbers that were obtained in the *in vivo* experiment are a relative enumeration of bacteria in the nasal cavity of each pig. Therefore, it is not possible to calculate the MOI for the *in vivo* experiment.

Results from the *in vitro* EOP experiments highlight the importance of developing a firm understanding of host bacteriophage relationships prior to commencing a bacteriophage therapy trial. While experiments in liquid culture indicated that the bacteriophage cocktail was highly effective against the V0608892/1 strain, plate cultures demonstrated that only one of the bacteriophages present (P68 present as 5% of the total pfu) killed the bacteria by lysis from within and that with greatly reduced efficiency compared to SAI653. It is possible that this relative lack of efficiency was responsible for the lack of efficacy observed in the *in vivo* and *ex vivo* experiments.

Additionally, this bacteriophage preparation was initially designed for use against human variants of MRSA. From the results from the *in vitro* experiment it was expected that these phages were equally effective against the MRSA-strain that was used in the *ex vivo* and *in vivo* experiments.

Bacteriophages were re-isolated from 5/8 piglets that received bacteriophage treatment using strain SAI653. However, the filtered sample suspension was ineffective against the bacteria that were re-isolated from the respective piglets, which were colonized with strain V0608892/1. After the bacteriophages were regrown—and stored at -80°C—they were found to be still effective against the MRSA isolates from the piglets they were isolated from. Most likely this was because the bacteriophages could not start a lysogenic infection in strain V0608892/1, but after enrichment on strain SAI763 were able to show lysis from without.

Alternatively, the presence of host-proteins may hamper the adherence of bacteriophages because of steric hindrance, since it is known that mammalian host-proteins cover the surface of bacteria [20,21]. However, only small amounts of mucus were observed in the scanning electron microscopic images that were taken of the nasal mucosa explants [17]. Also, the expression of bacterial proteins *in vitro* differs significantly from the expression *ex vivo* [22]. In this case there is either a lack of expression of the receptor in the bacteria when they are *in vivo*, or the receptor is masked by a surface component. Nonetheless, bacteriophages have already been successfully applied to reduce colonization of the intestines and caeca [23,24], which are also covered with mucus, but where the contents are mixed due to peristaltic action which would enhance the chances of bacteriophages reaching target cells. The gel formulation did not interfere with the phages (data not shown).

In conclusion, the MRSA strain was killed by the bacteriophages in the gel *in vitro*. However, MRSA reduction was not observed in the pig model or in the nasal mucosa explant model in contrast to mupirocin treatment. This may be due to differences in the experimental models, protein expression and binding *in vivo* and *ex vivo* or due to a lack of specific efficacy against the host strain used. It emphasises the need for extensive testing to gain a full understanding of phage-bacterial interactions *in vitro*—more than only observing lysis from without—before commencing *in vivo* studies.

## Supporting Information

S1 Table Effectivity of Phage K and P68 for different *Staphylococcus aureus* strains. (PDF)
