# Supplementary material for: The Effectiveness of Bacteriophages against Methicillin-Resistant Staphylococcus aureus ST398 Nasal Colonization in Pigs
Source: PLoS One. 2016 Aug 3;11(8):e0160242. doi: 10.1371/journal.pone.0160242 (PMC4972443; doi:10.1371/journal.pone.0160242)
Supplement: S1 Table — (PDF) [file pone.0160242.s001.pdf]

Supplementary Table 1

A **Effectivity of Phage K and P68 against human MRSA***

EMRSA group	15										16		13	16	15	15			16	
strain name	A554	A552	A256	A158	A782	A357	A542	A417	A25	A828	EMM 286	EMTI	A1093	MRSA 252	MRSA 509	PM25	PM77	PK78	PM64	PM79
Phage K	+	+	+	+	+	+	+	+	+	+	+	+	+	+	+	+	+	+	+	+
Phage P68	-	-	+	-	-	+	+	-	-	-	+	+	-	-	-	+	-	-	-	-

* Strains obtained from Heartlands Hospital, Birmingham; St. George University, London; Johns Hopskin hospital, London, United Kingdom

B **Effectivity of Phage K and P68 against pig MRSA**

strain name	DK-1	DK-2	DK-3	DK-4	DK-5	PIL77	PIL69	PIL74	PB40
ST type	n.a	n.a	n.a	n.a	n.a	n.a	ST398	ST398	ST9
Place and year of isolation	n.a	n.a	n.a	n.a	n.a	Denmark 2007	Italy 2008	Italy 2008	Hong Kong 2007
Phage K	-	-	+	-	-	+	+	-	+
Phage P68	+	+	-	-	+	-	+	+	+

* Strains obtained from the Staten Serum Institute, Copenhagen, Denmark

Propagation strain	SAI356
Phage K	+
Phage P68	+

C **Effectivity of Phage K and P68 against MRSA ST398***

strain	1	2	3	4	5	6	7	8	9	10
spa type	t011	t011	t899	t108	t567	t108	t1939	t108	t034	t1451
source	pig	pig	pig	pig	pig	human	pig	ıumar	calf	calf
Phage K	+	+	+	-	-	-	+	-	+	+
Phage 68	-	+	+	+	+	-	+	+	+	+

* Strains obtained from the Faculty of Veterinary Medicine, Utrecht, The Netherlands

(dark grey)	infectious: plaques formed at greatest dilution at
(medium grey)	infectious: less effectivity
(light grey)	Non-infectious but able to cause 'lysis from without'
(white)	Non-infectious and not be able to cause 'lysis from without'
